# Assessment of Model Accuracy in Eyes Open and Closed EEG Data: Effect of Data Pre-Processing and Validation Methods

**DOI:** 10.3390/bioengineering10010042

**Published:** 2022-12-29

**Authors:** Jamolbek Mattiev, Jakob Sajovic, Gorazd Drevenšek, Peter Rogelj

**Affiliations:** 1 Department of Information Technologies, Urgench State University, Khamid Alimdjan 14, Urgench 220100, Uzbekistan; 2 Department of Orthodontics, University Medical Centre Ljubljana, Hrvatski trg 6, 1000 Ljubljana, Slovenia; 3 Faculty of Medicine, University of Ljubljana, Korytkova 2, 1000 Ljubljana, Slovenia; 4 Faculty of Mathematics, Natural Sciences and Information Technologies, University of Primorska, Glagoljaška 8, 6000 Koper, Slovenia

**Keywords:** electroencephalography (EEG), machine learning, model validation, eyes closed, eyes open

## Abstract

Eyes open and eyes closed data is often used to validate novel human brain activity classification methods. The cross-validation of models trained on minimally preprocessed data is frequently utilized, regardless of electroencephalography data comprised of data resulting from muscle activity and environmental noise, affecting classification accuracy. Moreover, electroencephalography data of a single subject is often divided into smaller parts, due to limited availability of large datasets. The most frequently used method for model validation is cross-validation, even though the results may be affected by overfitting to the specifics of brain activity of limited subjects. To test the effects of preprocessing and classifier validation on classification accuracy, we tested fourteen classification algorithms implemented in WEKA and MATLAB, tested on comprehensively and simply preprocessed electroencephalography data. Hold-out and cross-validation were used to compare the classification accuracy of eyes open and closed data. The data of 50 subjects, with four minutes of data with eyes closed and open each was used. The algorithms trained on simply preprocessed data were superior to the ones trained on comprehensively preprocessed data in cross-validation testing. The reverse was true when hold-out accuracy was examined. Significant increases in hold-out accuracy were observed if the data of different subjects was not strictly separated between the test and training datasets, showing the presence of overfitting. The results show that comprehensive data preprocessing can be advantageous for subject invariant classification, while higher subject-specific accuracy can be attained with simple preprocessing. Researchers should thus state the final intended use of their classifier.

## 1. Introduction

In recent years, there have been many attempts to develop efficient classifiers of brain activity detected by electroencephalography (EEG), as EEG shows great promise as a tool for brain-computer interfaces (BCI) [1], the detection of emotion and epileptic disorder [2,3], cognitive load, Alzheimer’s disease and mild cognitive impairment [4,5]. To validate and verify the developed classifiers, the data acquired from subjects at rest with their eyes closed (EC) and with their eyes opened (EO) is often used. This is because the difference in the EEG signal in the two eye states is easily noticeable by visual inspection and therefore presents a relatively easy classification problem [6,7,8]. The most important distinguishing EEG characteristics of the EC and EO states are the presence (or absence) of eye-blinks and the change in the so-called α frequency power (7–13 Hz) of the detected brain waves [9,10].

As the EEG detects changes in electrical fields, generated by populations of firing neurons, it invariably detects the activity of motor nerve fibers as well. That is why the movement and blinking of the eyes in EO is very distinct in EEG recordings, as the muscles responsible for these movements are located directly below the recording electrodes of an EEG device [11]. In EEG research, such muscle activity in the recording is often considered to be an unwanted artifact, as it does not represent actual brain activity, but motor activity of facial muscles instead [12].

On the other hand, the increase in the α frequency band power during EC reflects the deactivation of the visual information processing system of the brain and its transition into a state of readiness [13,14,15]. The more chaotic, high frequency signal, with less regularities while the eyes are open, is thought to represent the complex processing of the information fed to the visual cortex via the optic nerves coming from the eyes and its integration into meaningful information that can be further processed and ultimately understood by brain areas involved in higher-order cognitive processes [15].

These two features illustrate a few fundamental characteristics of the EEG signals. The first is that there are many artifacts in EEG data, which confound the detection and interpretation of brain activity [11,12]. Next, certain conditions or actions can significantly influence the brain activity and thus markedly change the EEG signals. Last, the EO and EC conditions, for example, can be distinguished by either analyzing the artifacts (non-brain activity), by analyzing brain activity (i.e., the change in α frequency power) or by a mixture of both [12].

In previous research of EC and EO classification, studies developing the methods of classification of EEG for use in disease detection and BCI have often employed EC and EO EEG data as a form of validation of the developed classifiers. Contributing to the methodology of EEG signal classification, these have reached high classification accuracies of EC and EO data, and have thus been deemed efficient at solving at least simple classification problems pertaining to EEG data. However, while to the best of our knowledge impeccable in the process of classifier development, authors often treated EEG data as simple signals that require minimal preprocessing from their raw state, acquired from an EEG device [8,16,17,18,19,20,21,22,23,24,25].

This is a problem because naive data preprocessing methods leave artifacts in the EEG recording (or parts thereof) in the data [12], which can skew the results of classifier training, development, and generalizability to other tasks requiring the classification of brain activity that is not tied to the eyes being open or closed. The problem of contamination by artifacts is widely recognized in the field of neuroscience, where several data cleaning methods have been developed to allow for little signal loss and maximum removal of data artifact features. However, because of the strong necessity for human decision-making in the process, comprehensive cleaning of the data and building classifiers upon that is often impractical. As a result, simpler methods of EEG data preprocessing are frequently utilized.

We explored the effects of simple and comprehensive data preprocessing on classifier performance in EEG data and show:

- that interpersonal variability significantly affects the classification accuracy;
- data preprocessing methods significantly affect the classification accuracy;
- the resulting frequency-power feature dataset is significantly affected by interpersonal variability and data preprocessing.

## 2. Materials and Methods

### 2.1. Data Collection, Preprocessing, Feature Extraction and Dataset Generation

#### 2.1.1. Data Collection

The EEG data was collected using a 24-channel wireless EEG amplifier (Smarting Mobi, mBrainTrain LLC, Belgrade, Serbia) and saline-sponge caps (GT Gelfree-S3, Greentek Ltd., Wuhan, China). Electrode positions corresponded to the 10/20 system, placed on the Fp1, Fp2, AFz, F7, F3, Fz, F4, F8, C3, Cz, C4, CPz, T7, T8, TP9, TP10, P7, P3, Pz, P4, P8, POz, O1 and O2 positions. The ground electrode was placed at the AFz and the physical reference at the FCz position. The EEG was sampled at 512 Hz with a custom-created software package that allows for online quantitative EEG quality monitoring (BDI recorder, BrainTrip Ltd., Naxxar, Malta). A total of 50 participants were recruited for the experiment (9 of those males, average age 81.5 ± 8.9 SD years, 3 were left-handed). The recording session was divided into four 2-min segments. Two segments were recorded with eyes open and two with eyes closed, for a total of 8 min of EEG. There were breaks between each segment. During the recording, the subjects were seated and were instructed to simply relax and be mindful of their breathing. The study was approved by the Institutional review board for scientific investigations on human subjects of Science and Research Centre, Koper, Slovenia, approval number 0624-40/22.

#### 2.1.2. Data Preprocessing

In order to explore the effects of EEG preprocessing on classification results, we produced 11 datasets, which can be roughly divided into two groups, the comprehensively preprocessed datasets and the simply preprocessed datasets. All datasets were derived from the 50 EEG recordings provided by the partner company BrainTrip.

EEG data preprocessing for producing the comprehensively preprocessed datasets was done using several methods of EEG data preprocessing, to ensure that as little muscular activity as possible remains in the data. The procedure for data preprocessing was as follows:

1. Visual inspection and rejection of obvious electrode detachment/malfunction and other similar types of artifacts
2. Filtering (1–50 Hz)
3. Independent component analysis (ICA) decomposition
4. Rejection of artifact independent components
5. Channel automatic rejection by spectrum, ±3 SD outlier channels removed
6. Visual inspection and rejection of any remaining artifact data
7. Re-referencing to average
8. Blind source separation (BSS) correction of muscle artifacts
9. Interpolation of missing channels
10. Epoching of the data into 2 s long epochs

To produce the simply cleaned datasets, the following steps were taken, adapted and modified from [14], chosen because they represent a realistic pipeline for data preprocessing observed in previous work;

1. Visual inspection of the dataset to determine the extent of saturation with artifacts by inspecting the data for obvious electrode detachment/malfunction and other similar types of artifacts
2. Filtering (1–50 Hz)
3. Epoching of the data into 2 s long epochs
4. Removal of any epochs where the voltage observed exceeds ±100 μV

#### 2.1.3. Feature Extraction

After the preprocessing was carried out, all the datasets underwent feature extraction. Features of interest were limited to simple power-spectrum analysis using a Welch method Fast Fourier-Transform (FFT) on the 2 s long epochs of the data. The parameters of the Welch’s FFT were a Hann window 0.5 s in length, with 50% overlap of windows. The frequency bin powers encompassing the ranges of 4–7 Hz, 8–13 Hz, 13–30 Hz and 30–50 Hz were averaged to obtain the powers of the Θ, α, β and ɣ frequency bands. This was performed for the data obtained from each of the 24 electrodes that comprised the EEG datasets, resulting in 96 features and 1 binary nominal target variable.

In order to enable the comparison of our results with previous work, the features were selected based on previous studies, who frequently used them to classify EC and EO data [8,16,17,18,19,20,21,22,23,24,25]. Moreover, the frequency-power spectrum measures represent relatively simple, less computationally-intensive measures, whose generation can be implemented in real time and as such present salient features for classification of EEG data in various applications.

#### 2.1.4. Generated Datasets

**Figure 1:**
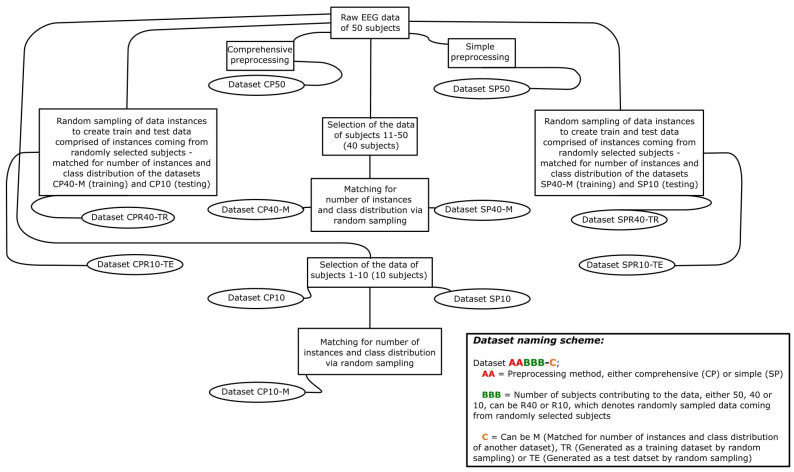
A schematic of the dataset generation process and the dataset naming scheme.

The purpose of creating these datasets (Figure 1) was to test the effects of sample size, interpersonal variability and preprocessing methods on the data produced for classification. Datasets CP40-M and CP10-M were matched to the size of the SP40-M and SP10 datasets, respectively, in order to eliminate possible effects of sample size and class distribution on the classification accuracies. The data samples of the comprehensively preprocessed datasets were randomly selected (random sampling) using the MATLAB random sampling function data sample without replacement. Detailed information about datasets is shown in Table 1.

### 2.2. Classification Models

In the present paper, several well-known models were used, that belong to different groups of methods for data classification, namely tree-based, rule-based, neural networks and linear models. The models were chosen, as they are commonly used to compare the effectiveness of newly developed models with established machine learning methods and can work with numeric features. More precisely, we evaluate the performance of following classification algorithms:

- Rule-based: JRip [26], this classifier implements a propositional rule learner, Repeated Incremental Pruning to Produce Error Reduction (also called RIPPER); PART [27], which unifies the two primary paradigms for rule generating: creating rules from decision trees and the separate-and-conquer rule learning technique by presenting an algorithm for inferring rules through iterative generation of partial decision trees.
- Tree-based: LMT (Logistic Model Trees) [28] classifier for building “logistic model trees”, which are classification trees with logistic regression functions at the leaves; J48 [29] classifier generates a pruned or unpruned decision tree and extract the rules for each path from root to the leave; Random Forest [30], which functions by building a large number of decision trees during the training phase and is an ensemble learning approach for classification, regression, and other tasks. The class that the majority of trees choose in a classification task is the random forest’s output.
- CNN-based models: The Temporal Convolutional Neural network (TCN) follows the work of Bai et al. [31] and consists of two convolutional blocks with 16 convolutional filters of size five each and dilation factors one and two, respectively. Each block contains two sets of dilated causal convolution layers with the same dilation factor, followed by normalization, leackyReLU activation, and spatial dropout layers.
- The Deep Neural Network (DNN) is a simple neural network consisting of three layers: the first layer is a dense layer with 12 units, and “relu” activation; the second layer is 8-unit dense layer (also with “relu” activation); the third layer is 1-unit dense layer with “sigmod” activation.
- Lazy: K-NN (K-Nearest Neighbor) [32], is an instance-based algorithm which selects k (K-NN can select the optimal k based on cross-validation) nearest neighbor and makes a prediction based on their class labels; KStar (instance-based model) [33], is an instance-based classifier, meaning that a test instance’s classification is based on the classification of training instances that are comparable to it as defined by some similarity function (entropy-based distance function).
- Linear models: ANN (Artificial Neural Network) [34], is an adaptable system that picks up new information by using interconnected nodes or neurons in a layered structure that attempts to simulate the structural organization of the human brain. A neural network may be trained to recognize patterns, classify data, and predict future occurrences since it can learn from data; SVM (Support Vector Machine) [34], Logistic Regression [35], classifiers for constructing and applying a multinomial logistic regression model with a ridge estimator.
- Ensemble classifiers: bagged trees and optimized ensemble classifier [32], ensemble classifiers meld results from many weak learners into one high-quality ensemble model.

We used the WEKA workbench [36] implementation of KStar, JRip, PART, Logistic Regression, LMT, J48 and Random Forest algorithms with the default parameters, as this enabled us to rapidly and simply train and test our classifiers. The main parameters of WEKA models are listed below:

1. Kstar: the global blending parameter was set to 20, and missing values were replaced with average column entropy curves.
2. Jrip: the “Pruning” method was applied, the minimum total weight of the instances in a rule was set to 2 and two optimization runs were used;
3. PART: the minimum number of instances per rule was 2, a common confidence factor 0.25 was used for pruning and the number of folds to determine the amount of data used for reduced-error pruning was set to 3;
4. Logistic Regression: The Ridge value (1 × 10^−8^) in the log-likelihood was applied as default;
5. LMT: the minimum number of instances at which a node is considered for splitting) was set to 15 and fast regression for using heuristic that avoids cross-validating the number of Logit-Boost iterations at every node was utilized;
6. J48: the default confidence factor (0.25) was used for pruning (smaller values incur more pruning. The subtree raising operation was utilized to improve the accuracy when pruning and the minimal number of instances per leaf was put to 2;
7. Random Forest: the number of trees in the random forest was set to 100 while the maximum depth of each tree was not limited.

For optimized classifiers, namely the Optimized Ensemble, Bagged Trees, K-NN, SVM and ANN, the MATLAB [34,37] software was employed. The parameters of optimized models were tuned automatically by Bayesian optimization procedures implemented in the MATLAB Statistics and Machine Learning Toolbox [34] (detailed information on parameter settings are provided in the Appendix A). The main parameters of WEKA models are as follows:

1. Optimized Ensemble: the optimized ensemble method of the most successful iteration was AdaBoost, with a maximum of 20 splits, 30 learners and 0.1 learning rate.
2. Bagged Trees: the learner type of the model was Decision Tree and the optimal number of split was 6602 with 100 learners;
3. K-NN: one neighbor was selected to make a decision and Euclidean metric was used to find the distance;
4. SVM: linear function was utilized in kernel mode and “One-vs-One” multiclass method was applied with 1 level of box constraint;
5. ANN: the number of fully connected layers (the size of each layer was 10) was set to 3 and “Relu” activation mode was used with 1000 iteration limit.

### 2.3. Statistics

Statistical testing was carried out in MATLAB 2022a (MathWorks, Natick, MA, USA) with a mixture of code available from the Statistics and Machine Learning Toolbox, MATLAB add-on EEGLAB and in-house developed code. For the comparison of EEG datasets, serial permutation *t*-tests were used with 10,000 permutations for the data derived from 10 subjects and 5000 permutations for the data derived from 50 subjects, due to computing intensity and hardware limitations. Testing was carried out in blocks, with each frequency band, data cleaning procedure and dataset size combination representing one block. For example, a comparison of the simply preprocessed and comprehensively preprocessed data in the Θ band would be comprised of a block of 24 comparisons, one for each electrode of the EEG recording. The obtained *p*-values would then be false discovery rate (FDR) corrected within this block, via the Benjamini-Yekutieli procedure. Next, the Bonferroni correction was applied to the FDR corrected p-values of each block, with the number of blocks serving as the number of comparisons to be corrected for. Altogether, 32 block comparisons were made. Post-correction two-sided threshold α = 0.05 was adopted throughout the analysis.

The statistical comparisons of the classifier hold-out accuracy and number of extracted data snippets was carried out with the use of chi-squared tests of independence, with the Bonferroni correction for multiple comparisons (the number of chi-squared tests carried out) applied. All *p*-values reported are post-correction *p*-values. The data fulfilled all the assumptions of the statistical tests used (namely the assumption of exchangeability in the case of permutation statistics and independence in the case of the chi-squared test).

The comparison of 10-fold cross-validation accuracies of the used classifiers was carried out in Microsoft Excel 2013 (The Microsoft Corporation, Redmond, WA, USA), by the use of two-tailed *t*-tests for independent samples. All *p*-values were Bonferroni-corrected and reported as such.

## 3. Results

First, the number of 2 s long data instances obtained from the simply and comprehensively preprocessed datasets was compared, to evaluate whether the two preprocessing methods are equivalent in the extraction of information from the raw EEG data. Of the possible 12,167 two-second-long data snippets that could have been extracted from the raw EEG data, 9481 were extracted using careful data cleaning procedures and 6603 were extracted with simple preprocessing procedures. In other words, comprehensive preprocessing resulted in 77.92% of the data being extracted, while simple preprocessing extracted only 54.27% of the data. This difference was statistically significant (chi-squared = 1519, *p* = 0).

Next, we evaluated the performance of 14 state-of-the-art classification models described in Section 2.2 on comprehensively and simply preprocessed EEG data based on the 10-fold cross-validation accuracies and the evaluation metrics of precision, recall and the F-measure [38]. Table 2 illustrates the experimental results of classification models on Dataset CP50 and Dataset SP50.

**Figure 2:**
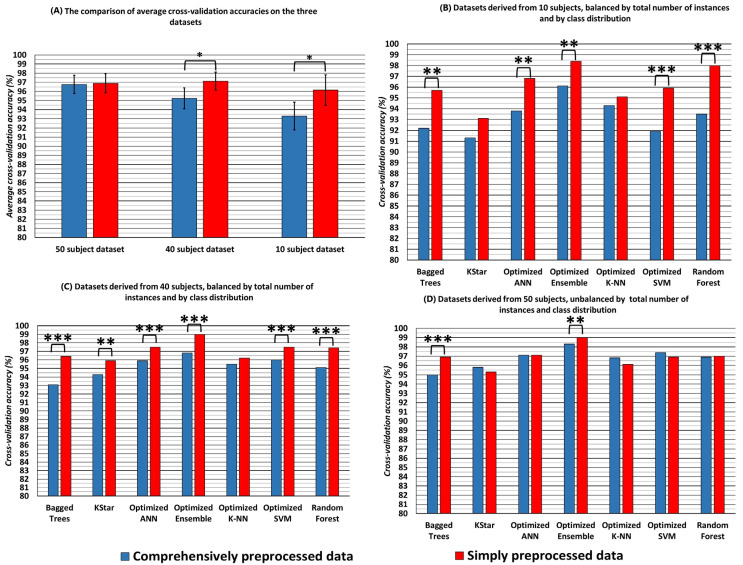
The average 10-fold cross-validation accuracies of the seven classifiers that obtained higher than 95% accuracy in 10-fold cross-validation. The accuracies were obtained by training the classifiers on the datasets and then averaged over all the used classifiers. Blue columns represent accuracies for the comprehensively preprocessed data, while red columns represent the accuracies for the simply preprocessed data. The * symbol denotes statistically significant difference in the average accuracy, at the level of *p* < 0.05, while the ** and *** symbols denote significance at the <0.01 and <0.001 level. The error bars in (**A**) represent 95% confidence intervals of the mean.

Table 2 shows that ensemble models constructed with Bayesian optimization procedures achieved the highest cross-validation accuracies, in both the comprehensively and simply preprocessed datasets (accuracies of 98.3 and 99%, respectively). Seven models achieved an accuracy of more than 95%, when cross-validated on both datasets. These models were the Kstar, Optimized K-NN, Optimized SVM, the Bagged trees model, Random forest, the Optimized ANN and the Optimized Ensemble model. All models that achieved more than 95% accuracy in the cross validation for either dataset were then compared. The average performance of these classifiers was also compared between the SP50 and CP50 datasets and between the CP10-M (Table A1) and SP10 (Table A2) and CP40-M (Table A3), SP40-M (Table A4) datasets. Figure 2 shows the results of these comparisons.

The results show that in the two datasets balanced for total size and class distribution, the classifiers trained on simply preprocessed data reached significantly higher accuracies in cross-validation than the classifiers trained on comprehensively preprocessed data (Figure 2, top left). When individual classifiers are compared in their performance on the three datasets (Figure 2, top right and bottom row), significant differences in accuracies can be observed for most of the classifiers in the two balanced datasets. Only the K-NN and Kstar models performed with no significant difference in the cross-validation, when trained on the naively and comprehensively preprocessed datasets. When trained on all available data, for the comprehensively preprocessed dataset and simply preprocessed dataset, unbalanced for total size and class distribution, the average cross-validation accuracy did not differ significantly. When individual classifiers are compared, however, the bagged trees and the optimized ensemble classifier achieved significantly higher accuracies when trained on the simply preprocessed data.

**Figure 3:**
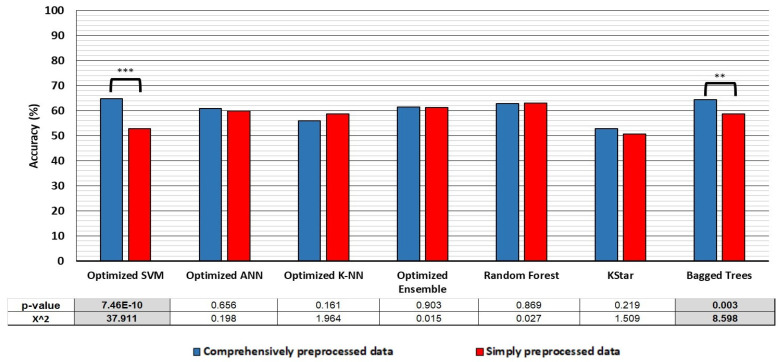
The performance of the seven models that achieved over 95% accuracy in the 10-fold cross validation testing in classification of the withheld comprehensively and simply processed datasets. The emboldened and grayed values in the table under the graph show statistically significantly different performances of a model on the two datasets. The *** symbol denotes statistical significance at the level of *p* < 0.001, while the ** symbol denotes statistical significance at the level of *p* < 0.01.

To explore how these cross-validation accuracies translate into effectiveness of the models to classify novel data, we have tested the models with the hold-out method. This means that we have trained the classifiers on the 40 subject dataset that was balanced for size and class distribution and then used these trained classifiers to classify the hold-out data (CP40-TR and SP40-TR datasets). The hold-out data was the data derived from ten subjects, balanced for size and class distribution (CP10-TE and SP10-TE datasets). The results are depicted in Figure 3, the detailed information is provided in Table A5.

**Figure 4:**
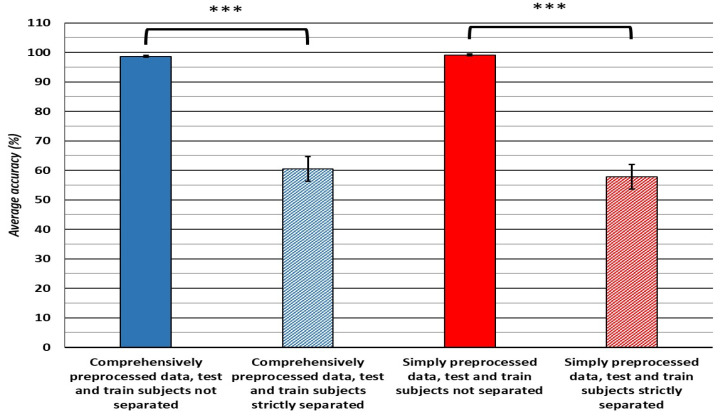
The comparison of hold-out validation accuracies on randomly sampled hold-out data and hold-out data belonging to the same subjects. The *** symbol denotes statistical significance at the level of *p* < 0.001. The error bars depict 95% confidence intervals for the mean.

The results show that, in contrast to cross-validation accuracies, there is either no significant difference in the accuracy of the classifiers when classifying novel data not included in the training dataset, or the classifiers trained on the comprehensively preprocessed data in fact performed better (the Optimized SVM and Bagged trees models) when faced with novel data (Figure 4). However, here it should be noted that when we tested the deep learning models on their ability to classify novel data, they achieved higher classification accuracies than other machine learning models, with the accuracies being 66.8% (DNN) and 60.6% (TCN), when tested on the comprehensively preprocessed datasets. When tested on the simply preprocessed datasets they achieved and 65.1% (DNN) and 71.4% (TCN) accuracy. Both methods reached the accuracy of ~85% in cross-validation testing (Table 2).

**Figure 5:**
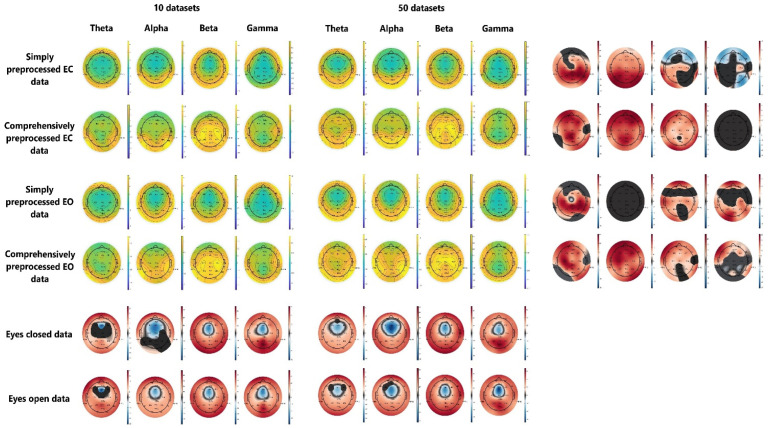
The comparison of frequency band powers by dataset size and data cleaning procedure. The data is organized to represent different frequencies by column (theta—Θ, alpha—α, beta—β, gamma—ɣ) and data cleaning and preprocessing procedure in rows (comprehensively and simply preprocessed data). The frequency powers are depicted in green and yellow colors, while the red-blue-and-black headplots depict *t*-values of permutation *t*-tests, comparing either simply and comprehensively preprocessed data (bottom) or the data derived from the first 10 with the data derived from the whole 50 subject dataset (right). The black patches on the red-blue-and-black headplots mark the areas where no significant differences were detected.

The last stream of our results consists of the examination of the created ten subject (SP10 and CP10) and 50 subject (CP50 and SP50) datasets. This was done in order to test whether dataset size and preprocessing method affect the final EEG analysis results. The results are depicted in Figure 5.

The results of the analysis of EEG data show that there are significant differences that can be observed, both as a result of preprocessing (Figure 5, bottom two rows) and as a result of dataset size (Figure 5, rightmost four columns). This means that preprocessing procedures meaningfully affect the features extracted from the EEG data, which can in turn further affect classifier performance. Moreover, the effects of dataset sample size were also pronounced, which shows that interpersonal variability additionally contributes to meaningful changes in EEG data, which carries its own implications for classifier development. If the number of compared electrodes that exhibit significant differences is considered, it can be concluded that the preprocessing method resulted in more widespread changes in the EEG signal than the dataset sample size.

## 4. Discussion

In the present paper, we examined the effects of EEG data preprocessing methods, interpersonal variability and sample size of subjects, which from the dataset is derived, on the classification accuracies of classifiers commonly used to classify the eye states, i.e., eyes open vs. eyes closed. We show that hold-out accuracy is significantly affected by separating the data by subject, not only on an instance-by-instance basis when constructing the training and test datasets. The data preprocessing method also affected the accuracy of some classifiers, both in cross-validation and hold-out testing. Moreover, we show that both the dataset size and preprocessing method produce significantly different features extracted from the EEG data.

The main findings of our study thus pertain to two main areas of classifier development;

1. **Classification accuracy:** the classifiers trained on simply preprocessed data performed better in the cross-validation, when the compared datasets are balanced for size and class distribution. However, the SVM and Bagged trees algorithms trained on comprehensively preprocessed data performed 12 and 5.7% better, respectively, in hold-out testing, with no differences in accuracy apparent in the cross-validation translating to differences observed in the hold-out accuracy in any of the classifiers.
2. **Dataset generation:** we have found that the EEG data that the classifiers train on differs significantly with regard to the preprocessing methods (and their strictness) used and with dataset sample size. Moreover, we have found that up to 23% more data can be extracted from the raw EEG recordings, when the data is carefully preprocessed.

With regard to classification accuracy, it is interesting that our study achieved very high classification accuracies of EC and EO data, with relatively simple preprocessing methods (in the case of simply preprocessed data), simple feature extraction methods and unmodified, off-the-shelf classification algorithms implemented in WEKA and MATLAB. For comparison with some previous studies that classified EC and EO resting state EEG data, please see Table 3 below.

Existing studies employing the data of more than ten subjects are compared first, as these studies used the best comparable number of subjects to our study. These studies [16,21,24] attained validation accuracies of 96.92%, 99.8% and 97.27%, with 10-fold cross-validation, 66–34% hold-out and 10-fold cross-validation, respectively. The number of subjects contributing to the datasets was 30 [24], 27 [21] and 109 [16]. In comparison to our attained 10-fold cross-validation accuracies of 98.3% for the comprehensively preprocessed dataset and 99% for the simply preprocessed one, our classification methods achieved higher accuracy than two of these studies. However, the feature selection was somewhat different in [16], but the results of [24] are directly comparable to the results of the present study. Additionally, all three studies can be understood as developing or testing classifiers for general-purpose use, with the authors of [16] explicitly stating so. Regardless, only the authors of [21] perform a hold-out method of testing, appropriate for testing the stability of the classifier in accuracy of the classification on data that significantly varies from subject to subject. Moreover, even when hold-out testing is used, it is unclear whether the withheld and the test data originate from different subjects or whether the instances of data are randomly assigned to each, without regard for subject origin. The highest accuracy of the hold-out testing presented in the current paper (Figure 3 and Figure 4) is either comparable to the one achieved in [21] (99.6% accuracy for simply and 99.1% accuracy for comprehensively preprocessed data) or vastly inferior (64.9% for the comprehensively and 63.1% for the simply preprocessed data), depending upon whether the data from different subjects is strictly separated or whether the instances are randomly assigned to the hold-out and test datasets, without regard for subjects of origin.

Next, the results of studies using similar feature extraction procedures as the present paper are examined. Six studies report using either the FFT or the wavelet transform to extract frequency-power features of EEG data [19,20,22,23,24,25], achieving 10-fold cross validation accuracies of 98.4, 97.96, 97, 96.92, 93 and 85.39%. The accuracies are largely comparable, among the studies and with the results of the present study, with the 85.39% accuracy of [25] standing out as being noticeably lower than the rest.

Furthermore, it is interesting that the studies employing no feature extraction, merely using the EEG signal as is, after limited preprocessing was employed [17,18,21] have reached high classification accuracies (99.8%, 95.2% and 98.5%, respectively). This could be interpreted as feature extraction being somewhat redundant for the classification of EEG data, although rigorous hold-out validation should be carried out before making this conclusion. The conclusion that not all important features of EEG signals can be extracted via the FFT could also be drawn from these results. This latter conclusion seems to be supported by the greater hold-out accuracy of the TCN model for the simply preprocessed data, which was concurrently the highest subject-invariant classification we were able to achieve (71.4%). The hold-out accuracies of all other methods were shown in Table A6.

On the topic of the classifiers used, the Random Forest, SVM and CNN types of classifiers were found to be the ones that most often produce best classification results, with two studies each reporting their results as being the best with the use of these classifiers to classify resting state EEG data into EC and EO. In our study, ensemble classifiers achieved better results than traditional rule-based classification methods, more precisely, the “Optimized Ensemble” classifier was the best model for classification using 10-fold cross-validation method while the “Optimized SVM” classifier obtained a slightly higher result on hold-out method.

From these previous results, we can conclude that the cross-validation accuracies achieved by the seven best classifiers on our datasets, ranging from 95 to 99%, are comparable to previous work on EC and EO classification from EEG signals. Our highest classification accuracy, achieved with simple preprocessing methods, simple feature extraction procedures and off-the-shelf classification algorithms is most likely due to our dataset being relatively large, being derived from 50 subjects. The fact that the dataset size affects classification accuracy is nothing new and has been reported multiple times previously. However, the extent to which a 5-fold increase in dataset size (and the number of subjects contributing to the data) affects the final extracted features of an EEG signal is still remarkable (Figure 5). This dataset size was nonetheless still likely too small to enable the full use of deep learning approaches, as reflected by relatively low cross-validation accuracies (Table 1).

Moreover, it is interesting that both the classifiers trained on the comprehensively preprocessed and simply preprocessed data achieved accuracies comparable to or exceeding previous studies on this topic, when evaluated by 10-fold cross validation. This could be interpreted as both sets of data being equally well suited for classification purposes, with the simply preprocessed data enabling even significantly higher (although not large, Figure 2) differences in classification accuracy.

However, the true differences in the suitability of the datasets for classification are revealed, when one compares the accuracies that classifiers trained on either dataset achieved in the hold-out validation process. There, the SVM and Bagged trees algorithms, out of the seven best classifiers that trained on the comprehensively preprocessed data have achieved significantly better (12 and 5.7%, respectively) classification accuracies than the classifiers trained on the simply preprocessed data, while the other five have shown no significant differences in classification accuracy.

To best explain this observation, the way in which the datasets were generated must be kept in mind. For the purpose of comparing the accuracies of the classifiers with the hold-out method, the training data consisted of the 2 s data instances belonging to the last 40 subjects, and was matched for size and overall class distribution. The test data consisted of the data of the first ten subjects, also matched for size and class distribution. This means that great care was taken to produce datasets, where test and training data are derived from different people, thus isolating the effects of interpersonal variability in the classification accuracy and presenting a realistic challenge to the trained classifiers, if the intention was to use them as general-purpose classifiers in real-world applications. When the data is matched for size and class distribution, the more comprehensive data preprocessing procedures can thus be concluded to be beneficial for controlling the effects of inter-subject variability, for at least the SVM and bagged trees classification algorithms. The reverse was true for the TCN model, however. There the problem of overfitting was more pronounced for the comprehensively preprocessed data, with the hold-out accuracy for the simply preprocessed data having been 10.8% greater. This could reflect the need for more data to be used in training for the deep learning approaches in general, with comprehensive preprocessing removing information useful to the machine learning model in the EC and EO classification process. An interesting contrast thus forms between the simpler and older machine learning approaches, such as bagged trees, and the more modern deep learning approaches to classification, where today’s methods rely on availability of big data to support the learning with more complex models, but the potential tradeoff seems to be a greater ability to discern subject invariant characteristics of EEG data. These findings should be more extensively verified and tested in future studies with more data utilized to enable the full drawing of such conclusions.

The overall result of the hold-out testing is important, as it shows that interpersonal variability greatly affects classification accuracy, which bears important implications for the development of future classifiers of EEG signals. This is further supported by the results of the hold-out testing without paying attention to the subject-specific origin of the datasets, that is using the data of the same subjects for training and testing the classifiers, by randomly sampling the same number of data instances as produced by the data of the last 40 subjects for training and randomly sampling the same number of data instances produced by the data of the first ten subjects for testing, without previously dividing it into two sets of subjects (Figure 4). This gives us both a training and testing dataset containing data instances of all 50 subjects. When the hold-out method of validation is used in this way, the classification accuracies of classifiers trained on either dataset exceed 90%, compared to ~60% accuracy on the hold-out testing with the data of different subjects, presenting a significant and large difference in the obtained accuracy. That interpersonal variability affects this data is further supported by results in Figure 5, where the features extracted from 50 subjects significantly differed from the ten-subject feature dataset on most electrode locations in most frequency bands, regardless of data preprocessing procedures used.

Moreover, these results show that the classifiers validated with cross-validation are prone to overfitting, with those trained on the comprehensively preprocessed data possibly being less prone to overfit, at least in the case of the SVM and bagged trees algorithms (Figure 3). Depending upon the intended final use of a classifier, these results bear important implications for the interpretation of the results of previous studies and future classifier development.

When the reporting of the accuracy of the developed and tested classifiers in previous work is examined, it becomes clear that ten-fold cross validation is the most popular method used to obtain accuracies, regardless of the final intended application of the classifier. Additionally, when the hold-out method is used, the procedure for the testing and training data generation is not sufficiently explained, being unclear whether the data for training and testing originates from different subjects or whether the data instances from all the subjects are mixed and then assigned to the testing and training datasets. This makes the degree of overfitting possibly present in the results unclear, which can be an issue.

The mentioned intended final use of the classifier is important here, as overfitting the data is not necessarily detrimental. For example, if one imagines that the goal of a project is to develop a BCI to be used by a single person, and that person only, then overfitting to the data of that person, to achieve the highest possible accuracy is appropriate. Conversely, if one wants to build a system for early detection of neurodegenerative diseases, for example, then special care should be taken not to overfit the data, as subject invariant classification models are crucial for these purposes.

With regard to most of the studies that have previously classified EC and EO in resting state EEG data developing classifiers for general-purpose use (Table 3, eight out of ten studies), meant for application without retraining the algorithms for each new user, we believe that these fall into the second group of applications, requiring subject invariant classification. Therefore, the reported accuracies, which consist mostly of the results of ten-fold cross-validation (Table 3, seven out of ten studies) might be skewed, when the intended use of the classifier is taken into account. However, only three studies out of ten explicitly state the final purpose of the classifier.

We propose that future studies clearly state what the intended final application of a classifier being developed is and that the data preprocessing and classifier validation methods are chosen based on whether overfitting to the data of a single subject (or a group of subjects) can be considered to be detrimental to the performance of the classifier. Moreover, in general purpose applications, where subject invariant classification is required, the 10-fold cross-validation alone might not be sufficient to validate the classifier to a satisfactory degree.

Perhaps less crucial, but an additional argument for comprehensive preprocessing of the data can be made when sample sizes are relatively small and the extraction of as many instances per dataset is necessary, as we show that up to 23% more data can be extracted from an EEG dataset when comprehensive preprocessing is employed. On the topic of dataset size, the large differences in the cross-validation and hold-out methods observed in this study could be interpreted as the sample size of subjects being too small to allow for the interpersonal variability being accounted for during the training of the classifiers. This could mean that more than 50 subjects are required to achieve classifiers that are interpersonally stable in accuracy, when using frequency band features of EEG datasets to classify the EO and EC conditions.

## 5. Conclusions and Future Work

To conclude, the present study shows that the data preprocessing methods of EEG significantly affect the final generated feature dataset. Furthermore, interpersonal variability of EEG data has been shown to significantly affect the classification accuracy of the EO and EC condition with the use of the hold-out validation method. When compared to the 10-fold cross-validation, the hold-out validation results differed significantly.

The methods applied in the present study are rather simple, however, when the broader scope of modern machine learning is considered. The effects observed here might not hold true for more specialized models, designed to deal with the issue of EC and EO classification specifically. However, as EC and EO classification is often used as the validating test of classifiers intended for other purposes, even more complex classifiers would greatly benefit in their validity, were they evaluated with interpersonal data variability in mind. Furthermore, to accurately and consistently evaluate modern machine learning methods, i.e., deep learning approaches, such as TCN, that are specialized for time-series classification, more data should be used. The amount of data needed for consistent performance and satisfactory training of these algorithms should be explored and the question of how much data is enough data answered. The issue of overfitting might also be somewhat ameliorated with a dataset of sufficient size being used.

With regards to these results and considerations, we propose that great care is taken when designing new classifiers, and to take into consideration whether the issue of overfitting to the data of the subject(s) which from the dataset is derived is detrimental to the final intended purpose of the classifier. If the answer to this question is yes, then comprehensive EEG data preprocessing, the usage of hold-out validation and sufficient sample size should be used to enable subject invariant classification.

## Figures and Tables

**Table 1:** Detailed information about the generated datasets.

Name	Number of Data Instances	Subjects Contributing to the Data	Number of Features	Class Distribution	
				EO	EC
CP50	9481	All 50	96	4711	4770
SP50	6603	All 50	96	3031	3572
CP40-M, SP40-M	5328 *	Subjects 11–50, 40 in total	96	2216	3112
CP10	1884	Subjects 1–10, 10 in total	96	940	944
SP10, CP10-M	1275 *	Subjects 1–10, 10 in total	96	460	815
CPR40-TR, SPR-40TR	5328 *	Various, randomly selected	96	2216	3112
CPR10-TE, SPR10-TE	1275 *	Various, randomly selected	96	460	815

* Abbreviations; CP—comprehensively preprocessed, SP—simply preprocessed, 50, 40 or 10, the size of the datasets (same number of instances as the datasets derived from 50, 40 or 10 subjects), M—matched to simply preprocessed data of the same size, TR—training data, TE—test data. Instance numbers marked with the * symbol; instances are selected separately for each dataset, although the sizes of the datasets are the same.

**Table 2:** The performance of the classifiers on *CP50* and *SP50* datasets.

Models	Accuracy (%)		Precision (%)				Recall (%)				F-Measure (%)			
			EO		EC		EO		EC		EO		EC	
	CP50	SP50	CP50	SP50	CP50	SP50	CP50	SP50	CP50	SP50	CP50	SP50	CP50	SP50
**Optimized Ensemble**	98.3	99.0	98.4	94.9	98.2	98.5	98.1	98.2	98.4	95.8	98.2	96.5	98.3	97.1
**Optimized SVM**	97.4	96.9	98.0	96.4	96.8	97.3	96.8	96.9	98.0	97.0	97.4	96.6	97.4	97.1
**Optimized ANN**	97.1	97.1	95.6	95.0	98.6	98.9	98.6	98.6	95.7	95.9	97.1	96.8	97.1	97.4
**Random Forest**	96.9	97.0	97.6	96.7	96.2	97.4	96.1	97.8	97.6	96.0	96.8	97.2	96.9	96.7
**Optimized K-NN**	96.8	96.1	95.1	93.5	98.4	98.3	98.3	97.9	95.3	94.7	96.7	95.6	96.8	96.5
**KStar**	95.8	95.3	97.9	94.1	93.9	96.9	93.5	97.5	98	92.8	95.7	95.8	95.9	94.8
**Bagged Trees**	95	96.9	93.2	94.9	96.7	98.5	96.5	98.2	93.6	95.8	94.8	96.5	95.1	97.1
**LMT**	92.6	93.6	95.4	92.0	90.1	95.7	89.4	96.6	95.7	90.1	92.3	94.2	92.8	92.8
**PART**	90.5	90.9	92.9	90.2	88.4	92.0	87.5	93.5	93.4	88.0	90.2	91.8	90.8	89.9
**J48**	88.4	88.4	90.7	86.4	86.4	90.7	85.5	91.3	91.3	85.5	88	88.8	88.8	88.0
**DNN**	86.8	86.3	90.0	91.2	84.1	82.8	79.7	78.4	92.1	91.1	84.5	84.3	87.9	86.8
**TCN**	84.7	83.2	89.6	88.0	81.0	79.4	78.5	76.5	91.0	89.7	83.7	81.8	85.7	84.2
**JRip**	85.9	87.5	87.4	87.2	84.6	87.9	83.8	90.1	88.1	84.4	85.5	86.6	86.3	88.1
**Logistic Regression**	79.4	75.9	77.4	65.4	81.6	84.9	79.9	78.5	76.2	74.3	79.9	71.4	78.8	79.2

Abbreviations; SVM—support vector machine, ANN—artificial neural network, K-NN—K-nearest neighbors, LMT—logistic model tree, PART—partial decision tree, EO—eyes open, EC—Eyes closed.

**Table 3:** Results of previous studies classifying EC and EO states from resting state EEG recordings.

Study	Subjects (# of Samples)	EEG	Preprocessing	Features Extracted	Best Classifier	Accuracy	Validation	Final Purpose
[14]	9 (257)	16	The data was preprocessed by filtering (1–40 Hz) and removing epochs exceeding ±100 μV of amplitude	FFT, all available frequency bins used as features (total number not stated).	SVM	97%	10-fold CV	The study aimed to identify the brain’s resting status using short-length EEG epochs using both linear and nonlinear features derived from EEG. Aimed at general-purpose applications. *
[15]	10 (112,128)	32	Bandpass filtering in the delta, theta, alpha and beta bands.	Delta, theta, alpha and beta band energies (128 features total).	Random Forest	85.39%	10-fold CV	This method exploits high spatial information acquired from the Emotiv EPOC Flex wearable EEG recording device and examines successfully the potential of this device to be used for BCI wheelchair technology. Aimed at targeted or person-specific final applications. *
[23]	30 (1800)	33	EEG data was first re-referenced to average, then bandpass-filtered between 1 and 50 Hz	Features were extracted based on the FFT in 6 (delta, theta, alpha, beta, gamma and all) different frequency bands, per 6 electrodes in 4 ROIs and 30 trials for each condition (8.640 features total).	CNN	96.92%	10-fold CV	Developing a more practical and reliable ear-EEG based application related to eye state classification. Aimed at general-purpose final applications. *
						90.81%	Test-retest	
[16]	27 (829,494)	19	“Clean” sections of data, without artifacts, were chosen by an expert.	Single electrode data points (19 features)	K-NN (k = 1)	99.8%	hold-out (66%/34% split)	Predicting eye states using EEG recordings in a real-time system, interpreting classification rules for gaining insight into EEG data. Aimed at general-purpose final applications. *
				10 data points of an EEG recording (190 features)	Random Forest	96.6%	hold-out (66%/34% split)	
	27 (82,836)		“Clean” sections of data, without artifacts, were chosen by an expert. The data was filtered in the 1–40 Hz range and a notch filter eliminating line noise was applied.	Single electrode data points (19 features)	K-NN (k = 1)	99.4%	hold-out (66%/34% split)	
				10 data points of an EEG recording (190 features)	Random Forest	96.5%	hold-out (66%/34% split)	
[17]	109 (3270)	64	EEG data was preprocessed by applying low pass Butterworth filtering with the cut off frequency of 40 Hz	Six RQA (recurrence quantification analysis)-based measures (recurrence rate, determinism, entropy, laminarity, trapping time, and longest vertical line) had been extracted from 64 EEG channels based on a genetic algorithm (384 features total).	Logistic Regression	97.27%	10-fold CV	Automated classification of EEG signals into eyes-open and eyes-close states. The development of the practical applications for performing daily life tasks. Aimed at general-purpose final applications.
[18]	1 (14,980)	Not stated.	No preprocessing.	The features (14 features) of EEG data were extracted based on the wavelet transform (Final number of features not stated).	Deep factorization machine model (FM: +LSTM	93%	10-fold CV	The diagnosis of fatigue by detecting eye openness status. Aimed at general-purpose final applications.
[19]	1 (14,980)	14	Clustering into dissimilar groups by Self-organizing map (SOM). Classification performed within each cluster.	No feature extraction, raw signal used.	Deep Belief Network (DBN)	95.2%	10-fold CV	The study investigates eye state identification using EEG signals. Aimed at general-purpose final applications. *
[20]	1 (15,181)	14	Dataset cleaned by removing the missing values as well as outliers based on the “Isolation Forest’’ technique	No feature extraction, raw signal used.	Hypertuned SVM	98.5%	10-fold CV (70/30 split)	Medical appliances capable of classifying various bodily states, drug effects etc. Aimed at general-purpose final applications.
[21]	10 (Not stated)	64	No preprocessing.	Mean value of each sliding window, asymmetry and peakedness, maximum and minimum, complex logarithm of sample covariance matrix, 10 most energetic FFT components, all windows also inherit features of previous windows that are different from the current ones (Variable number of total features for each window.)	CNN	97.96%	10-fold CV	The approach is dynamically applicable to BCI devices of higher resolution and problems other than the frontal lobe activity classification. Aimed at targeted or person-specific final applications. *
						83.3%	“Leave-one-out” CV	
[22]	1 (14,980)	14	First, the Independent Component Analysis (ICA) algorithm is employed to remove the artifacts from EEG data, then the data is Fast Fourier Transformed.	Fast Fourier Transform, time analysis, time-frequency analysis using the STFT, and time-frequency-space analysis extracted characteristics of data were chosen as features based on the results of Principle Component Analysis (14 features).	EBPTA	98.94%	10-fold CV	The eye status categorization from brain activity signals and its application to real time applications. Aimed at general-purpose final applications. *
This paper	50 (6603)	24	Visual inspection of the dataset to assess its usability, filtering (1–50 Hz), epoching of the data into 2 s long epochs, removal of any epochs where the voltage observed exceeds ±100 μV.	Welch FFT acquired frequency bin powers encompassing the ranges of 4–7 Hz, 8–13 Hz, 13–30 Hz and 30–50 Hz were averaged to obtain the powers of the Θ, α, β and ɣ frequency bands. This was done for the data obtained from each of the 24 electrodes that comprised the EEG datasets, resulting in 96 features and 1 binary nominal target variable.	Ensemble Classifier	99%	10-fold CV	Final purpose is the evaluation of the effects of preprocessing and validation methods on classifier accuracy.
	50 (6603)				Random Forest	63.1%	Hold-out (40 subjects/10 subjects)	
	50 (9481)		Visual inspection and rejection of obvious electrode detachment/malfunction and other similar types of artifacts, filtering (1–50Hz), independent component analysis (ICA) decomposition, rejection of artifact independent components, channel automatic rejection by spectrum, ±3 SD outlier channels removed, visual inspection and rejection of any remaining artifact data, re-referencing to average, blind source separation (BSS) correction of muscle artifacts, interpolation of missing channels, epoching of the data into 2 s long epochs.		Ensemble Classifier	98.3%	10-fold CV	
	50 (6603)				SVM	64.9%	hold-out (40 subjects/10 subjects)	

* Studies marked with *; the final application aimed at is not explicitly stated by the authors of the paper, but is inferred from the conclusions or statements authors make in their article. General-purpose—meant for use by a wide group of people, without personalization and adjustment of the classifiers. Targeted/person-specific purpose—meant for use by a specific person or a small group, classifier is meant to be adjusted and retrained for each individual user. Abbreviations; SVM—support vector machine, CNN—convolutional neural network, K-NN—K-nearest neighbors, FM—Factorization Machine, LSTM—Long Short-Term Memory, EBPTA—Evolutionary Back Propagation Training Algorithm, FFT—Fast Fourier Transform, CV—cross validation.

**Figure A1:**
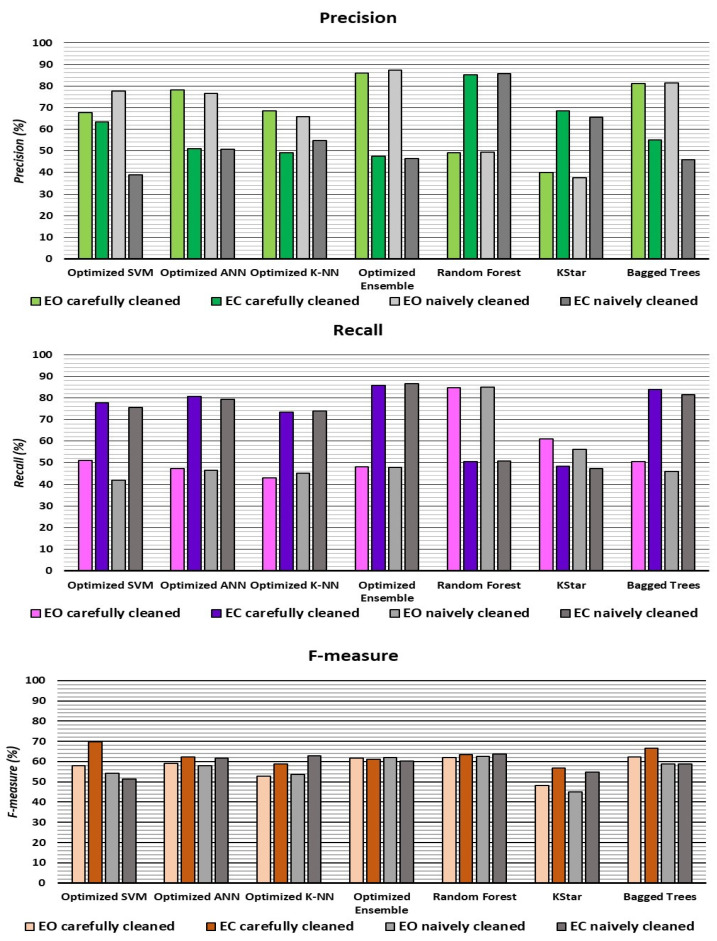
The precision, recall and F-measure for the comprehensively preprocessed and simply preprocessed data, with the classifiers trained on the data derived from 40 subjects and tested on the data derived from the withheld data of ten subjects.

## Data Availability

The data used in this study is proprietary, it will be provided in edited version via the figshare repository during the review process, pending partner company approval.

## Appendix A

**Table A1:** The performance of the classifiers on *Dataset CP10-M*.

Dataset	Accuracy	Precision		Recall		F-Measure	
		EO	EC	EO	EC	EO	EC
**Optimized Ensemble**	96.1	93.7	97.4	95.4	96.5	94.5	96.9
**Optimized ANN**	93.8	91.7	95.0	91.1	95.3	91.4	95.1
**Optimized K-NN**	94.3	83.3	97.1	94.5	94.2	95.6	95.4
**Random Forest**	93.5	94.6	93.0	87.0	97.2	90.6	95.0
**Optimized SVM**	91.9	90.0	93.0	87.9	94.3	88.9	93.6
**Bagged Trees**	92.2	85.7	96.0	92.3	92.2	88.9	94.1
**KStar**	91.3	89.4	92.3	86.1	94.2	87.7	93.3
**LMT**	88.9	85.7	90.6	83.0	92.1	84.3	91.4
**J48**	86.6	80.9	89.9	82.2	89.1	81.6	89.5
**PART**	86.9	83.7	88.7	79.3	91.3	81.5	90.0
**Logistic Regression**	86.9	82.9	89.0	80.2	90.7	81.5	89.8
**JRip**	86.2	80.5	89.5	81.5	88.8	81.0	89.2

**Table A2:** The performance of the classifiers on *Dataset SP10*.

Dataset	Accuracy	Precision		Recall		F-Measure	
		EO	EC	EO	EC	EO	EC
**Optimized Ensemble**	98.4	98.9	97.4	98.5	98.0	98.7	97.7
**Random Forest**	98.0	97.5	98.9	99.4	95.4	98.4	97.1
**Optimized ANN**	96.8	97.5	95.4	97.4	95.6	97.4	95.5
**Optimized SVM**	95.9	97.8	92.6	95.9	95.9	96.8	94.2
**Bagged Trees**	95.7	97.7	92.2	95.7	95.7	96.7	93.9
**Optimized K-NN**	95.1	97.5	90.7	94.9	95.4	96.2	92.9
**LMT**	94.0	94.5	93.0	96.2	90.0	95.3	91.5
**KStar**	93.1	92.6	94.1	96.9	86.3	94.7	90.0
**PART**	92.3	93.1	90.8	95.0	87.6	94.0	89.2
**J48**	91.2	92.5	88.8	93.9	86.5	93.2	87.7
**JRip**	90.4	92.1	87.2	92.9	85.9	92.5	86.5
**Logistic Regression**	88.2	90.0	84.7	91.7	82.0	90.8	83.3

**Table A3:** The performance of the classifiers on *Dataset CP40-M*.

Dataset	Accuracy	Precision		Recall		F-Measure	
		EO	EC	EO	EC	EO	EC
**Optimized Ensemble**	96.8	94.9	98.1	97.3	96.4	96.1	97.2
**Optimized SVM**	96.0	92.8	98.2	97.3	95.1	94.9	96.6
**Optimized ANN**	95.9	93.0	98.1	97.2	95.1	95.1	96.6
**Optimized K-NN**	95.5	92.2	97.9	96.9	94.6	94.5	96.2
**Random Forest**	95.1	96.8	93.9	91.1	97.9	93.9	95.9
**KStar**	94.3	95.8	93.3	90.3	97.2	93.0	95.2
**Bagged Trees**	93.1	88.1	96.7	94.9	92.0	91.4	94.3
**LMT**	90.9	93.4	89.5	84.3	95.7	88.6	92.5
**PART**	89.6	90.3	89.2	84.1	93.6	87.1	91.3
**JRip**	87.5	86.9	87.9	82.3	91.2	84.5	89.5
**J48**	87.1	88.5	86.3	79.3	92.6	83.7	89.4
**Logistic Regression**	80.8	77.3	83.3	76.4	84.0	76.8	83.7

**Table A4:** The performance of the classifiers on *Dataset SP40-M*.

Dataset	Accuracy	Precision		Recall		F-Measure	
		EO	EC	EO	EC	EO	EC
**Optimized SVM**	97.5	97.6	97.4	96.4	98.3	96.9	97.8
**Optimized ANN**	97.5	95.2	99.2	98.9	96.7	97.0	97.9
**Optimized K-NN**	96.2	92.0	99.1	98.6	94.6	95.2	96.8
**Optimized Ensemble**	99.0	98.3	99.4	99.2	98.8	98.7	99.1
**Random Forest**	97.4	99.1	96.3	94.6	99.4	96.8	97.8
**KStar**	95.9	98.7	94.2	91.4	99.2	94.9	96.6
**Bagged Trees**	96.4	92.4	99.2	98.7	94.8	95.4	96.9
**LMT**	95.4	98.2	93.6	90.5	98.8	94.2	96.2
**PART**	93.5	94.9	92.5	89.1	96.6	91.9	94.5
**J48**	92.7	94.2	91.8	88.0	96.1	91.0	93.9
**JRip**	90.3	89.9	90.6	86.4	93.1	88.1	91.8
**Logistic Regression**	81.7	79.4	83.3	75.7	86.0	77.5	84.6

**Table A5:** The performance of models on evaluation measures for “comprehensively” and “simply” preprocessed datasets based on train/test split method: 40 subjects (various, randomly selected) for train/ten subjects (various, randomly selected) for test. CP4/1: CPR40-TR and CPR10-TE; SP4/1: SPR40-TR and SPR10-TE.

Models	Accuracy		Precision				Recall				F-Measure			
			EO		EC		EO		EC		EO		EC	
	CP4/1	SP4/1	CP4/1	SP4/1	CP4/1	SP4/1	CP4/1	SP4/1	CP4/1	SP4/1	CP4/1	SP4/1	CP4/1	SP4/1
**Optimized SVM**	98.6	98.7	97.4	98.0	99.3	99.1	98.7	98.5	98.5	98.9	98.0	98.2	98.9	99.0
**Optimized ANN**	99.1	99.1	98.5	98.7	99.5	99.4	99.1	98.9	99.1	99.3	98.8	98.8	99.3	99.3
**Optimized K-NN**	98.9	99.5	97.4	99.1	99.8	99.6	99.6	99.3	98.5	99.5	98.5	99.2	99.1	99.5
**Optimized Ensemble**	99.1	99.6	98.7	99.3	99.4	99.8	98.9	99.6	99.3	99.6	98.8	99.4	99.3	99.7
**Random Forest**	98.3	99.2	97.8	99.1	98.5	99.3	97.4	98.7	98.8	99.5	97.6	98.9	98.7	99.4
**KStar**	98.4	99.1	98.7	99.3	98.2	99.0	96.7	98.3	99.3	99.6	97.7	98.8	98.7	99.3
**Bagged Trees**	98.5	99.0	97.4	98.5	99.1	99.3	98.5	98.7	98.5	99.1	97.9	98.6	98.8	99.2

**Table A6:** The performance of models on evaluation measures (“Precision”, “Recall” and “F-measure”) for “comprehensively” and “simply” preprocessed datasets based on train/test split method: 40 subjects for train/10 subjects for test. CPH40/10: CP40-M and CP10; SPH40/10: SP40-M and SP10.

Models	CPH40/10							SPH40/10						
	Accuracy	Precision		Recall		F-Measure		Accuracy	Precision		Recall		F-Measure	
		EO	EC	EO	EC	EO	EC		EO	EC	EO	EC	EO	EC
**Optimized SVM**	64.9	67.6	63.3	51.0	77.6	58.1	69.7	52.9	77.6	38.9	41.8	75.5	54.3	51.3
**Optimized ANN**	60.8	78.3	50.9	47.4	80.6	59.1	62.4	59.9	76.5	50.6	46.6	79.2	57.9	61.7
**Optimized K-NN**	56	68.5	49.0	43.1	73.3	52.9	58.7	58.7	65.9	54.7	45.1	74.0	53.6	62.9
**Random Forest**	62.8	49.1	85.3	84.6	50.6	62.1	63.5	63.1	49.4	85.7	85.0	50.8	62.5	63.8
**KStar**	52.8	39.9	68.6	60.9	48.3	48.2	56.7	50.6	37.5	65.6	56.1	47.2	44.9	54.9
**Bagged Trees**	64.5	81.1	55.1	50.5	83.8	62.2	66.5	58.8	81.5	46.0	46.0	81.5	58.8	58.8
**TCN**	60.6	59.7	61.7	64.6	56.6	62.1	59.0	71.4	86.4	65.1	50.9	91.9	64.1	76.2
**DNN**	66.8	64.2	66.9	71.3	63.1	67.6	64.9	65.1	74.8	61.4	50.1	81.1	60.0	69.9
